# Associations between non-insulin-based insulin resistance indices and heart failure prevalence in overweight/obesity adults without diabetes mellitus: evidence from the NHANES 2001–2018

**DOI:** 10.1186/s12944-024-02114-z

**Published:** 2024-04-27

**Authors:** Di-yu Cui, Chao Zhang, Yi Chen, Gang-zhen Qian, Wan-xiang Zheng, Zhi-hui Zhang, Yu Zhang, Ping Zhu

**Affiliations:** Department of Cardiovascular Medicine, Center for Circadian Metabolism and Cardiovascular Disease, Southwest Hospital, Third Military Medical University (Army Medical University), 30 Gaotanyan Street, Shapingba District, Chongqing, 400038 China

**Keywords:** Triglyceride-glucose index, Triglyceride-to-high-density lipoprotein cholesterol ratio, Heart failure, Overweight/obesity

## Abstract

**Background:**

The triglyceride glucose (TyG) index and triglyceride-to-high-density lipoprotein cholesterol (TG/HDL-C) ratio are recognized as simple non-insulin-based insulin resistance indices. Our study aimed to explore the relationship between these two indicators and heart failure (HF) in overweight or obesity individuals without diabetes.

**Methods:**

This cross-sectional study selected 13,473 participants from the National Health and Nutrition Examination Survey (NHANES) 2001–2018 dataset. Weighted multivariable logistic regression and subgroup analysis were employed to evaluate the relationships between TyG index, TG/HDL-C ratio, and HF prevalence, respectively. Additionally, smooth curve fitting was utilized to analyze the dose–response relationships.

**Results:**

A total of 13,473 obesity or overweight people without diabetes were included in this study through screening, among whom 291 (2.16%) had comorbid HF. The results of multivariable logistic regression suggested that the highest TyG index (OR = 2.4, 95% CI = 1.4–4.2, *p* = 0.002) and the highest TG/HDL-C ratio (OR = 1.2, 95% CI = 1.1–1.3, *p* < 0.001) both increased the prevalence of HF, especially in the non-Hispanic population. Dose–response relationships suggested nonlinear relationships between these two indicators and HF.

**Conclusion:**

Our study demonstrated that elevated TyG index and TG/HDL-C ratio were closely associated with the prevalence of HF, and both exhibited nonlinear relationships with HF prevalence in overweight/obesity adults without diabetes. Based on these findings, additional prospective studies are needed for further validation.

**Supplementary Information:**

The online version contains supplementary material available at 10.1186/s12944-024-02114-z.

## Introduction

With economic development, obesity has become an important social problem in many countries. In March 2023, the World Obesity Federation declared that by 2035, more than 4 billion people worldwide will be affected by overweight or obesity, constituting more than half of the global population [1]. Obesity causes insulin resistance (IR), and the resulting accumulation of insulin promotes fat synthesis, thus establishing a vicious cycle [2]. Overweight and obesity are closely related to many common diseases, including diabetes, hypertension, and coronary heart disease (CHD), and is also more common in heart failure (HF) patients, with the prevalence of overweight and obesity in HF patients reaching 40.3% and 31.2%, respectively [3].

As a manifestation of metabolic disorders, IR is strongly associated with HF and has been confirmed by numerous studies to be an independent risk factor for the development of HF. In addition, IR is prevalent in the HF population [4–6]. The main accurate and reliable method for assessing IR is the euglycemic insulin clamp. However, its clinical application is challenging due to technical complexity. The most commonly utilized alternative is the homeostatic model assessment of insulin resistance (HOMA-IR) index. Despite its widespread use, this method is not commonly employed in routine clinical practice due to factors such as complexity, time consumption, and cost. Currently, the triglyceride glucose (TyG) index and triglyceride/high-density lipoprotein (TG/HDL-C) ratio are considered convenient, inexpensive, and reliable non-insulin-based methods for estimating IR and have been shown to have high sensitivity [7, 8]. Numerous studies have assessed the relationship between these two indicators and the risk of developing cardiovascular diseases, including HF, and have consistently that they are strongly associated [9, 10]. We observed differences in the results of subgroup analyses among overweight/obesity patients with or without diabetes in studies investigating the association between IR and HF [11–13]. While existing studies have predominantly focused on analyzing risk factors for HF in overweight/obesity individuals with diabetes, there is a notable gap in research pertaining to overweight/ obesity populations without diabetes [14–16]. However, it is crucial to separately evaluate this population for HF risk factors because of the distinct disease progression of HF in populations without diabetes compared to that of populations with diabetes. Therefore, we explored the relationship between IR and HF using the TyG index and the TG/HDL-C ratio, two non-insulin-dependent indices of IR, using data from the National Health and Nutrition Examination Survey (NHANES) database. The objective of this study was to effectively guide subsequent relevant prospective studies by identifying the risk factors for HF in overweight/obesity patients without diabetes, thereby providing clinical evidence for risk stratification and individual management.

## Methods

### Study population

**Fig. 1:**
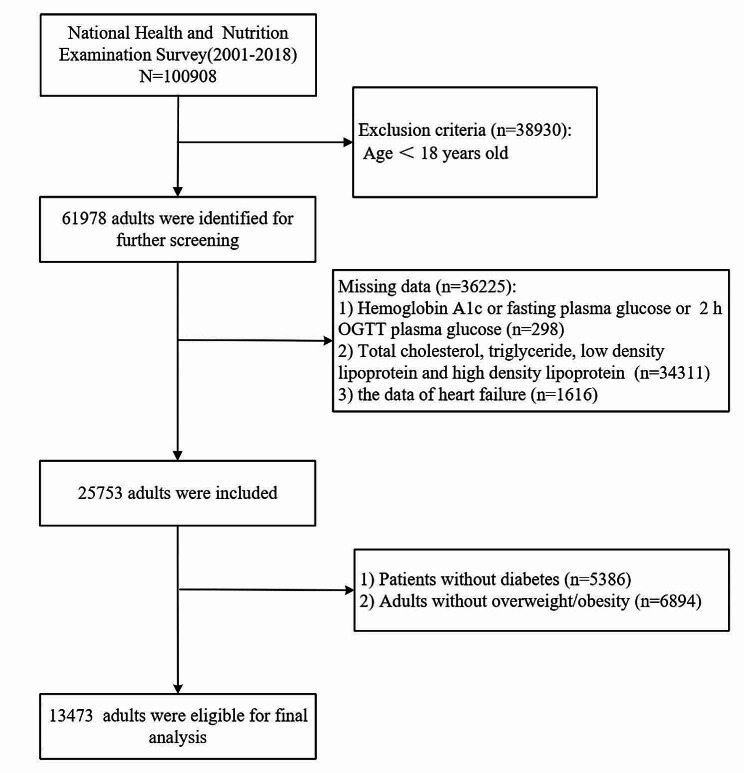
Flow chart of participants selection from the NHANES 2001–2018

The National Health and Nutrition Examination Survey (NHANES) is a major epidemiological survey conducted by the National Center for Health Surveys (NCHS) of the U.S. Department of Health and Human Services (HHS) to assess the health and nutritional status of the U.S. population. For this study, we extracted population data (*n* = 100,908 individuals) from the 2001–2018 NHANES database. We excluded participants under 18 years of age (*n* = 38,930), as well as those with missing glucose (*n* = 298), blood lipid (*n* = 34,311), or HF questionnaire (*n* = 1616) data. We excluded 5386 patients with diabetes and 6894 individuals who had normal weight. A total of 13,473 adults were ultimately included in the analysis (Fig. 1).

### Diabetes and overweight/obesity status

Diabetes status [17] was defined if the patients fulfilled at least one of the following criteria: (1) Fasting plasma glucose (FPG) ≥ 7 mmol/L, (2) HbA1c ≥ 6.5%, (3) 2-h plasma glucose during the OGTT ≥ 11.1 mmol/L, or (4) self-reported physician-diagnosed diabetes.

Overweight/obesity [18] was defined as a body mass index (BMI) ≥ 25 kg/m^2^.

#### TyG index and TG/HDL-C ratio

The TyG index was calculated using the following formula: Ln [fasting TG (mg/dL) × FPG (mg/dL)/2]. The TG/HDL-C ratio was calculated as TG (mg/dL) divided by HDL (mg/dL).

We classified the participants into four groups (Q1, Q2, Q3, and Q4) according to the quartiles of the TyG index and TG/HDL-C ratio, and the Q1 group served as the reference.

#### HF

In the NHANES, HF data were collected via a health questionnaire administered during a personal interview. In the self-report questionnaire, an affirmative answer to the following question was considered to indicate HF status: “Have you ever been told by a doctor or other health professional that you had congestive heart failure?”

#### Covariable definitions

We selected the following basic population characteristics for their potential to influence the relationships of the TyG index and TG/HDL-C ratio indices with HF in a population with obesity but without diabetes: age, sex, ethnicity (including Mexican American, Other Hispanic, Non-Hispanic White, Non-Hispanic Black, or Other), HbA1c, FPG, systolic blood pressure (SBP), diastolic blood pressure (DBP), albumin, creatinine, TC, HDL, LDL, TG, smoking status, stroke, CHD and impaired fasting glucose (IFG).

The definition of IFG according to the World Health Organization (WHO) is based on an FPG in the range of 6.1–6.9 mmol/L.

### Statistical analysis

During the data analysis, we considered the weighting of the NHANES database. We categorized the total population into those with and without HF and analyzed the basic characteristics of the patients in both categories. Continuous variables are presented as the mean ± standard deviation or median (interquartile range) depending on their normal distribution, while categorical variables are presented as counts and percentages (%). A weighted linear regression model was used for continuous variables, and weighted chi-square tests were performed to analyze differences between categorical variables. The associations of the TyG index and the TG/HDL-C ratio with HF prevalence among adults with overweight/obesity but without diabetes were assessed by survey-weighted logistic regression analysis, and odds ratios (ORs) and 95% CIs were calculated via two models. Model 1 was adjusted for age, sex and ethnicity, and Model 2 was adjusted for age, sex, ethnicity, SBP, DBP, albumin, creatinine, LDL, TC, smoking status, CHD prevalence and stroke prevalence.

We then performed sensitivity analyses by stratified analysis and interaction tests with subgroups to test for interactions and effects of relevant confounding factors, including age (< 60 and ≥ 60), sex, ethnicity, SBP (< 140 mmHg and ≥ 140 mmHg), smoking status, stroke status, CHD status and IFG.

Dose–response relationships of the TyG index and TG/HDL-C ratio with the prevalence of HF were observed using generalized additive model (GAM) and fitted curve methods. If nonlinearity was detected, we first utilized smoothed curve fitting to assess whether the independent variable was partitioned into intervals. Then, we apply segmented regression, which employs separate line segments to fit each interval. A log-likelihood ratio test is conducted using a single-line model versus a segmented regression model to determine the presence of a threshold. The maximum likelihood is determined based on the model, and the inflection points of the connected segments are identified using a two-step recursive approach. Finally, we assessed the difference between the results of the two-part logistic regression model.

All the data analyses in this study were conducted using R software (http://www.R-project.org) and Empower (version 5.0; www.empowerstats.com; X&Y Solutions).

## Results

### Baseline characteristics

This study ultimately included 13,473 adults with a mean age of 47.82 ± 17.00 years, 47.57% of whom were male. Compared with the non-HF population, the HF population was older and had higher HbA1c levels, FPG levels, TyG index, TG/HDL-C ratio and creatinine levels as well as a greater prevalence of comorbidities such as stroke, CHD and IFG. The detailed results are presented in Table 1.

**Table 1:** Baseline Characteristics of participants

	Overall *N* = 13,473	HF *N* = 291	Non-HF *N* = 13,182	*P*-value
Age (years)	47.82 ± 17.00	65.05 ± 14.53	47.44 ± 16.85	< 0.001
Males	6409 (47.57%)	144 (49.48%)	6265 (47.53%)	0.966
Race/Ethnicity				0.003
Mexican American	2382 (17.68%)	16 (5.50%)	2366 (17.95%)	
Other Hispanic Non-Hispanic White Non-Hispanic Black Other Race	1378 (10.23%) 5688 (42.22%) 2895 (21.49%) 1128 (8.37%)	19 (6.53%) 173 (59.45%) 70 (24.05%) 13 (4.47%)	1359 (10.31%) 5515 (41.84%) 2825 (21.43%) 1115 (8.46%)	
HBA1C (%)	5.41 ± 0.01	5.59 ± 0.39	5.46 ± 0.38	< 0.001
BMI (kg/m2)	29.90 (27.30-33.94)	30.70 (27.38–35.55)	29.90 (27.30-33.94)	0.034
FPG (mmol/L)	94.35 ± 9.86	97.83 ± 10.49	94.27 ± 9.83	< 0.001
TC (mg/dl)	195.60 ± 40.34	179.13 ± 43.29	195.96 ± 40.20	0.005
LDL (mg/dl)	118.08 ± 34.59	101.61 ± 35.64	118.45 ± 34.48	< 0.001
HDL (mg/dl)	52.50 ± 14.56	51.25 ± 16.91	52.53 ± 14.51	0.165
TG (mg/dl)	108.00 (76.00-155.00)	121.00 (83.00-174.50)	107.00 (75.00-154.00)	0.021
TyG index	8.54 ± 0.53	8.68 ± 0.52	8.53 ± 0.53	< 0.001
TG/HDL-C ratio	2.12 (1.36–3.34)	2.68 (1.50-4.00)	2.11 (1.36–3.33)	0.006
Stroke	374 (2.78%)	51 (17.53%)	323 (2.45%)	< 0.001
SBP (mmHg)	117.92 ± 22.66	120.65 ± 29.21	117.86 ± 22.49	0.411
DBP (mmHg)	67.52 ± 14.48	63.00 ± 16.87	67.62 ± 14.41	0.003
Albumin(g/dl)	4.16 ± 0.35	4.05 ± 0.36	4.16 ± 0.35	< 0.001
Creatinine(mg/dL)	0.88 ± 0.38	1.12 ± 0.49	0.87 ± 0.38	< 0.001
Smoke	5845 (43.38%)	169 (58.08%)	5676 (43.06%)	< 0.001
IFG	3226 (23.94%)	95 (32.65%)	3131(23.75%)	< 0.001
CHD	393 (2.92%)	98 (34.27%)	295 (2.24%)	< 0.001

Data are presented as number (%) or mean ± standard deviation (SD) or Median (Q1-Q3)

For continuous variables: *P*-value was by survey-weighted linear regression. For categorical variables: *P*-value was by survey-weighted Chi-square test

TyG index, the triglyceride glucose index; TG/HDL-C ratio, triglyceride to high-density lipoprotein cholesterol ratio; BMI, body mass index; SBP, systolic blood pressure; DBP, diastolic blood pressure; LDL, low density lipoprotein; HDL, high density lipoprotein; TC, total cholesterol; TG, triglyceride; IFG, impaired fasting glucose; CHD, coronary heart disease; FPG, Fasting plasma glucose

### Associations of the TyG index and TG/HDL-C ratio with HF prevalence

In the multivariable logistic regression, both the TyG index and the TG/HDL-C ratio were divided into quartiles. After adjusting for age, sex, race, SBP, DBP, albumin, creatinine, LDL, TC, smoking status, CHD status, and stroke status in Model II, both the TyG index and TG/HDL-C ratio exhibited positive association with HF prevalence (TyG: OR = 2. 4, 95% CI: 1.4–4.2, *p* = 0.002; TG/HDL-C: OR = 1.2, 95% CI: 1.1–1.3, *p* < 0.001). The HF prevalence was greater in the Q4 subgroup than in the Q1 subgroup (TyG: OR = 3. 2, 95% CI: 1.7–5.9, *p* < 0.001; TG/HDL-C: OR = 3.4, 95% CI: 1.9-6.0, *p* < 0.001). The results are shown in Table 2.

**Table 2:** Relationship between TyG index, TG/HDL-C ratio and the prevalence of HF using logistic regression models

Outcome	Crude Model ^a^			Model I ^b^			Model II ^c^	
	OR (95%CI)	*P*-value		OR (95%CI)	*P*-value		OR (95%CI)	*P*-value
TyG index	2.2 (1.5, 3.2)	< 0.001		2.1 (1.3, 3.3)	0.003		2.4 (1.4, 4.2)	0.002
TG/HDL-C ratio	1.2 (1.1, 1.3)	< 0.001		1.2 (1.1, 1.3)	< 0.001		1.2 (1.1, 1.3)	< 0.001
TyG (quartile)								
Q1(0.49–1.36)	1(Reference)			1(Reference)			1(Reference)	
Q2(1.36–2.12)	1.0 (0.6, 1.9)	0.895		0.9 (0.5, 1.6)	0.680		1.0 (0.5, 1.9)	0.895
Q3(2.12–3.34)	1.8 (1.1, 2.9)	0.0023		1.5 (0.9, 2.5)	0.147		1.7 (0.9, 3.1)	0.086
Q4(3.34–9.72)	2.7 (1.7, 4.3)	< 0.001		2.3 (1.4, 3.8)	0.002		3.2 (1.7, 5.9)	< 0.001
TG/HDL-C (quartile)								
Q1(7.33–8.17)	1(Reference)			1(Reference)			1(Reference)	
Q2(8.17–8.53)	1.0 (0.5, 1.7)	0.890		1.0 (0.6, 1.9)	0.908		1.2 (0.6, 2.3)	0.624
Q3(8.53–8.90)	1.7 (1.0, 2.9)	0.039		1.8 (1.1, 3.1)	0.022		2.0 (1.1, 3.7)	0.022
Q4(8.90–9.70)	2.2 (1.4, 3.4)	< 0.001		2.8 (1.7, 4.6)	< 0.001		3.4 (1.9, 6.0)	< 0.001

^**a**^**Crude Model** adjusted for none

^**b**^**Model I** adjusted for age, sex and ethnicity

^**c**^**Model II** adjusted for age, sex, ethnicity, systolic blood pressure (mmHg), diastolic blood pressure (mmHg), albumin(g/dl), creatinine (mg/dL), low density lipoprotein (mg/dl), total cholesterol (mg/dl), smoke statue, stroke and coronary heart disease

**Fig. 2:**
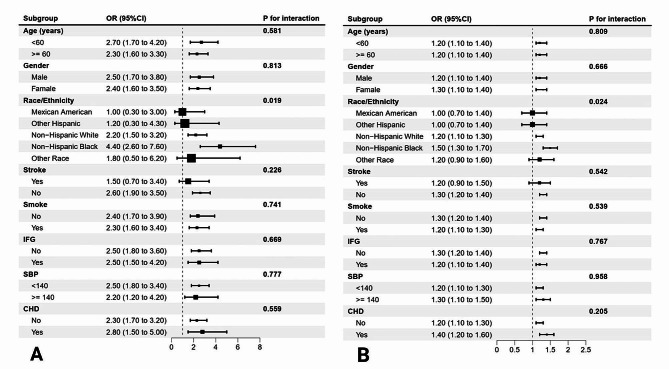
Subgroup analysis of the association between the prevalence of HF and TyG index **(A)**, TG/HDL-C ratio **(B)**. Adjusted for age, sex, ethnicity, systolic blood pressure (mmHg), diastolic blood pressure (mmHg), albumin(g/dl), creatinine (mg/dL), low density lipoprotein (mg/dl), total cholesterol (mg/dl), smoke statue, stroke and CHD, except the stratified factor itself. IFG, impaired fasting glucose; CHD, coronary heart disease; SBP, systolic blood pressure

We performed subgroup analyses (Fig. 2) of age (< 60 and ≥ 60 years), sex, race, SBP (< 140 mmHg and ≥ 140 mmHg), smoking status, stroke status, CHD status and IFG, and the associations of the TyG index and TG/HDL-C ratio with HF prevalence were stable. However, ethnicity influenced the associations between the TyG index and the TG/HDL-C ratio and HF prevalence (TyG: p for interaction = 0.019; TG/HDL-C: p for interaction = 0.024). The TyG index and TG/HDL-C ratio were strongly associated with HF prevalence in non-Hispanic populations, whereas there was no clear association in other races.

### Dose–response relationships

**Fig. 3:**
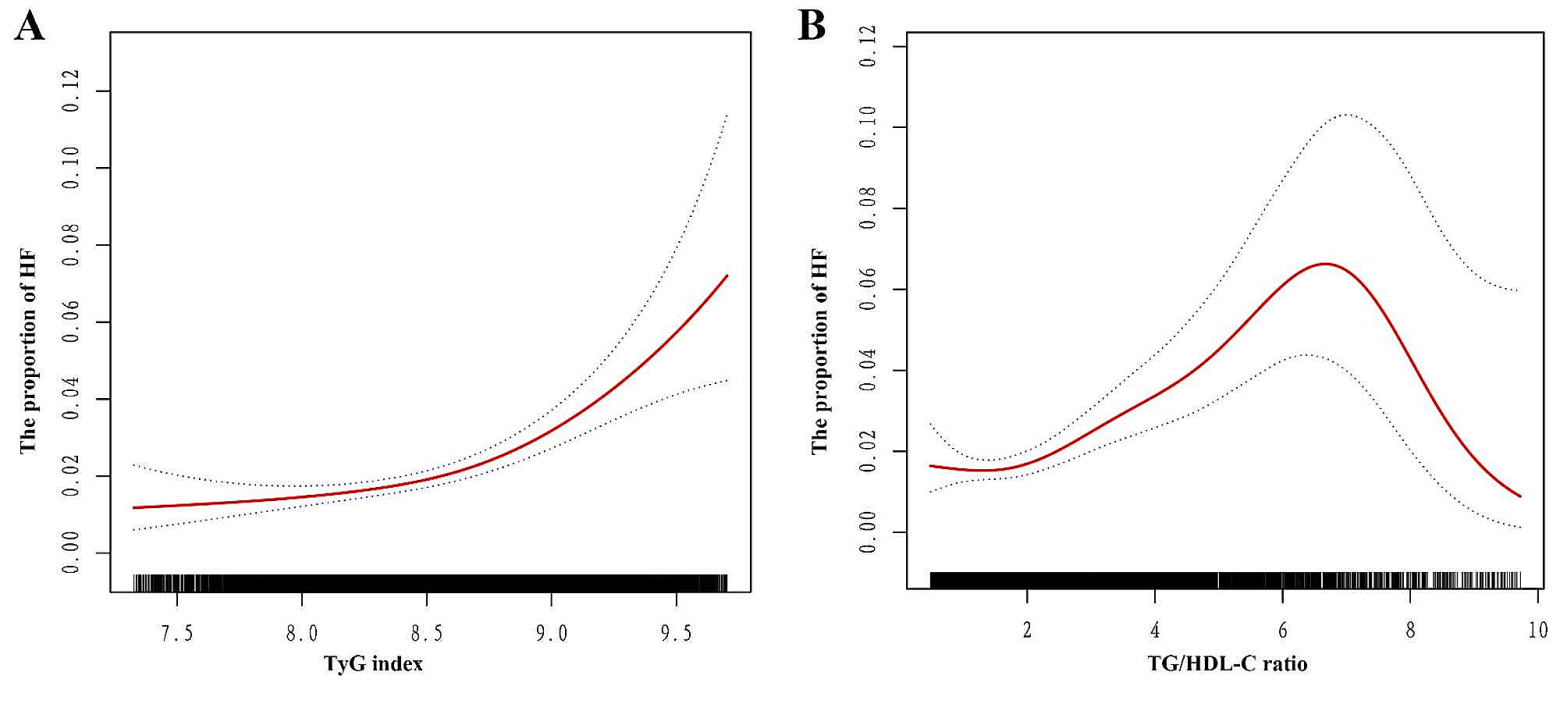
Smooth curve fitting using Generalized Additive Model (GAM) to evaluate the nonlinear relationship between TyG index (A), TG/HDL-C ratio (B) and the proportion of HF. The red solid line represents the probability of HF occurrence and the blue dotted line represents the 95% CI curve

In addition, the results of the smoothed curve-fitting analysis confirmed a nonlinear positive association between the TyG index and the prevalence of HF (Fig. 3A), with an inflection point of 8.5 according to the threshold effect analysis (Table 3). The prevalence of HF increased with a TyG index greater than 8.5 (OR = 4.0, 95% CI = 2.4–6.4; *p* < 0.001). Smoothed curve-fitting analysis also suggested that the TG/HDL-C ratio was nonlinearly related to the prevalence of HF (Fig. 3B), with an inflection point of 6.2 according to threshold effect analysis (Table 4).

**Table 3:** Threshold effect analysis of TyG index on HF prevalence using a two-part logistic regression model

TyG index	Adjusted OR (95%CI), *P*-value
Model I	
Fitting by the standard linear model	2.4 (1.8, 3.3) < 0.001
Model II	
Inflection point	8.5
< 8.6	1.1 (0.6, 2.7) 0.721
> 8.6	4.0 (2.4, 6.4) < 0.001
Log likelihood ratio	0.012

**Table 4:** Threshold effect analysis of TG/HDL-C ratio on HF prevalence using a two-part logistic regression model

TG/HDL-C ratio	Adjusted OR (95%CI), *P*-value
Model I	
Fitting by the standard linear model	1.2 (1.2, 1.3) < 0.001
Model II	
Inflection point	6.2
< 6.2	1.4 (1.2, 1.5) < 0.001
> 6.2	0.7 (0.4, 1.1) 0.082
Log likelihood ratio	0.001

## Discussion

This large-sample cross-sectional study based on data from the NHANES database was conducted to assess the associations of the TyG index and TG/HDL-C ratio with the prevalence of HF in an overweight/obesity population without diabetes. Within this population, we observed a strong association between an elevated TyG index and TG/HDL-C ratio and a high prevalence of HF. Subgroup analyses and interaction tests indicated that this association was present in non-Hispanic populations, with no clear association observed in other racial groups.

In the present study, the TyG index and TG/HDL-C ratio were utilized to assess IR, and both indices had a nonlinear relationship with the prevalence of HF; threshold analyses identified inflection points of 8.5 and 6.2 for the TyG index and TG/HDL-C ratio, respectively. The TyG index has been shown to associate well with the results of the euglycemic insulin clamp [7]. Some of these studies have indicated that a higher TyG index is a risk factor for HF prevalence [19, 20]. Hou et al. [21] reported that the prevalence of HF increased rapidly with an increasing TyG index exceeding 8.91, a threshold similar to our findings. The TG/HDL-C ratio, proposed as an indicator of atherosclerosis by Gaziano et al. [22], is associated with a greater proportion of small dense LDL particles, which are highly atherogenic [23]. Consequently, this indicator has been recognized as a predictor for atherosclerotic cardiovascular disease [24]. With further research, it has been discovered that the TG/HDL-C ratio can serve as a simple parameter for evaluating IR and is a useful indicator for identifying metabolic syndrome, particularly in overweight individuals [25–27]. This study is the first to explore the usefulness of the TG/HDL-C ratio in an HF population, thereby expanding the application of the TG/HDL-C ratio in heart disease populations. We hope that more studies will follow to further substantiate our conclusions.

The associations of diabetes, obesity, and overweight with HF are robust and often reciprocal. The current study revealed that, within the population without diabetes, non-insulin-based insulin resistance indices (the TyG index and TG/HDL-C ratio) were associated with a greater prevalecne of HF in individuals with obesity, and the results remained stable in subgroup analyses of individuals with abnormal glucose tolerance. Diabetes is a recognized metabolic factor that elevates the prevalence of HF, and other metabolic abnormalities, such as IR, similarly contribute to an increased prevalence of HF in obesity individuals without diabetes mellitus. IR is strongly associated with the risk, severity, and poor prognosis of HF. Using the HOMA-IR index, the ARIC Large Community Cohort Study revealed that IR was associated with the development of HF [28]. Metabolic syndrome and the development of HF are closely related to comorbid cardiovascular disease in patients without diabetes, and some of these effects are mediated by the HOMA-IR index [29]. IR results in disruptions to myocardial carbohydrate and lipid metabolism, mitochondrial and endoplasmic reticulum dysfunction, and endothelial dysfunction, ultimately leading to cardiac systolic and diastolic dysfunction [30]. On the other hand, hyperinsulinemia causes water and sodium retention, excites the sympathetic nervous system [31], and may induce cardiac remodeling, leading to impaired structural, functional [32], and metabolic changes that drive the progression of HF pathogenesis.

The presence of HF also predicts the development of IR, with glycemic abnormalities being highly prevalent in HF patients (43% of patients). Additionally, the prognosis for HF patients without diabetes is likely to be worse than that for patients with normal blood glucose and insulin levels [33–35]. An observational cohort study of 58,056 patients without diabetes revealed that HF patients had a significantly greater risk of developing diabetes later in life [36]. The increased risk of new-onset diabetes in patients with HF is primarily due to the progression of advancing IR [37, 38], the mechanisms of which are intricate. HF induces heightened sympathetic nerve activity, and this increased sympathetic nervous system activity can result in decreased insulin responsiveness, glucose utilization, and β-cell insulin secretion by impacting vasodilatory tone, free fatty acid levels, and oxidative stress [39]. Increased aldosterone impairs insulin secretion and sensitivity, which is a key factor in the development of diabetes [40]. Plasma aldosterone is strongly associated with BMI or an index of IR [41]. Angiotensin II (ANGII) can stimulate the blood vessel wall to generate reactive oxygen species (O2^−^), leading to inflammation, apoptosis, and reduced insulin formation and secretion [42]. Additionally, angiotensin II receptor 2 promotes pancreas-driven oxidative stress, apoptosis, and fibrosis, thereby affecting insulin production [43].

Subgroup analyses revealed that the effect of IR on HF prevalence was meaningful in non-Hispanic populations, regardless of sex (black or white). Differences in cardiovascular prevalence between non-Hispanic populations and other racial groups have been reported to some extent [44–46]. However, the exact reasons for these differences are currently unknown, and additional studies are needed to explore this further.

### Limitations

Some limitations of this study should be considered. First, we were unable to establish a causal association between the TyG index or TG/HDL-C ratio and the onset of HF due to the cross-sectional design. Future multicenter prospective studies are necessary to confirm potential causal associations and identify respective inflection points. Second, the inclusion criteria for HF patients were self-reported, which may have led to an underestimation of the prevalence of HF. Third, although we adjusted for several potential confounders in our analysis, the results may still be influenced by other unknown factors. Finally, this study was based on a U.S. population and may not be generalizable to other countries.

## Conclusion

Our study showed that an elevated TyG index and TG/HDL-C ratio were strongly and nonlinearly associated with a greater prevalence of HF in individuals with overweight or obesity but without diabetes.

### Electronic supplementary material

Below is the link to the electronic supplementary material.

Supplementary Material 1

## Data Availability

No datasets were generated or analysed during the current study.

## Abbreviations

CI: confidence interval
OR: odds ratio
