# Supplementary material for: Associations between non-insulin-based insulin resistance indices and heart failure prevalence in overweight/obesity adults without diabetes mellitus: evidence from the NHANES 2001–2018
Source: Lipids Health Dis. 2024 Apr 27;23:123. doi: 10.1186/s12944-024-02114-z (PMC11055335; doi:10.1186/s12944-024-02114-z)
Supplement: Supplementary file 1 — Supplementary Material 1 [file 12944_2024_2114_MOESM1_ESM.docx]

**Supplementary Table 1.** Baseline Characteristics of participants

|  | TG/HDL-C ratio  Q1 | Q2 | Q3 | Q4 | P-value |
| --- | --- | --- | --- | --- | --- |
| Age (years) | 47.21 ± 17.41 | 48.19 ± 17.23 | 48.80 ± 17.27 | 47.42 ± 16.18 | 0.347 |
| Males | 1196 (35.50%) | 1458 (43.30%) | 1720 (51.07%) | 2035 (60.40%) | <0.001 |
| Race/Ethnicity |  |  |  |  | <0.001 |
| Mexican American | 429 (12.73%) | 595 (17.76%) | 674 (20.15%) | 684 (20.45%) |  |
| Other Hispanic  Non-Hispanic White  Non-Hispanic Black  Other Race | 269 (8.00%)  1264 (37.52%)  1182 (35.08%)  224 (6.65%) | 338 (10.04%)  1356 (40.27%)  823 (24.44%)  255 (7.57%) | 371 (11.02%)  1483 (44.03%)  538 (15.97%)  301 (8.94%) | 400 (11.87%)  1585 (47.05%)  352 (10.45%)  348 (10.33%) |  |
| HBA1C (%) | 5.40 ± 0.38 | 5.46 ± 0.39 | 5.49 ± 0.39 | 5.50 ± 0.38 | <0.001 |
| BMI (kg/m2) | 29.10 (26.80-32.89) | 29.65 (27.20-33.61) | 30.40 (27.60-34.30) | 30.80 (27.92-34.90) | <0.001 |
| FPG (mg/dl) | 91.95 ± 9.43 | 93.77 ± 9.41 | 95.31 ± 9.94 | 96.43 ± 10.03 | <0.001 |
| TC (mg/dl) | 186.78 ± 37.17 | 192.77 ± 39.22 | 196.93 ± 41.11 | 205.87 ± 41.42 | <0.001 |
| LDL (mg/dl) | 108.24 ± 30.83 | 118.54 ± 32.77 | 122.16 ± 35.46 | 124.58 ± 36.33 | <0.001 |
| HDL (mg/dl) | 65.02 ± 13.92 | 54.82 ± 10.92 | 48.57 ± 9.63 | 41.12 ± 7.74 | <0.001 |
| TG (mg/dl) | 62.00 (52.00-74.00) | 92.00 (80.00-106.00) | 125.00 (109.00-143.00) | 193.00 (163.00-234.0) | <0.001 |
| Stroke | 97 (2.88%) | 100 (2.97%) | 87 (2.58%) | 90 (2.67%) | <0.001 |
| SBP (mmHg) | 116.54 ± 22.20 | 118.54 ± 22.64 | 118.12 ± 23.11 | 118.54 ± 22.65 | <0.001 |
| DBP (mmHg) | 65.96 ± 14.19 | 67.47 ± 14.09 | 67.46 ± 14.78 | 69.21 ± 14.52 | <0.001 |
| Albumin(g/dl) | 4.13 ± 0.33 | 4.14 ± 0.34 | 4.14 ± 0.36 | 4.20 ± 0.36 | <0.001 |
| Creatinine(mg/dL) | 0.85 ± 0.33 | 0.87 ± 0.26 | 0.88 ± 0.37 | 0.91 ± 0.52 | <0.001 |
| Smoke | 1236 (36.69%) | 1388 (41.22%) | 1567 (46.53%) | 1654 (49.09%) | <0.001 |
| IFG | 550 (16.33%) | 742 (22.04%) | 902 (26.78%) | 1032 (30.63%) | <0.001 |
| CHD | 92 (2.73%) | 74 (2.20%) | 113 (3.36%) | 114 (3.38%) | 0.130 |
| HF | 58 (1.72%) | 59 (1.75%) | 75 (2.23%) | 99 (2.91%) | <0.001 |

Data are presented as number (%) or mean ± standard deviation (SD) or Median (Q1-Q3)

For continuous variables: P-value was by survey-weighted linear regression. For categorical variables: P-value was by survey-weighted Chi-square test.

TG/HDL-C ratio, triglyceride to high-density lipoprotein cholesterol ratio; BMI, body mass index; SBP, systolic blood pressure; DBP, diastolic blood pressure; LDL, low density lipoprotein; HDL, high density lipoprotein; TC, total cholesterol; TG, triglyceride; IFG, impaired fasting glucose; CHD, coronary heart disease; FPG, Fasting plasma glucose.

**Supplementary Table 2.** Baseline Characteristics of participants

|  | TyG index  Q1 | Q2 | Q3 | Q4 | P-value |
| --- | --- | --- | --- | --- | --- |
| Age (years) | 43.90 ± 16.69 | 48.14 ± 17.32 | 49.77 ± 16.91 | 49.89 ± 16.50 | <0.001 |
| Males | 1321 (40.47%) | 1606 (47.68%) | 1638 (48.61%) | 1844 (54.72%) | <0.001 |
| Race/Ethnicity |  |  |  |  | <0.001 |
| Mexican American | 451 (13.81%) | 585 (17.37%) | 661 (19.61%) | 685 (20.33%) |  |
| Other Hispanic  Non-Hispanic White  Non-Hispanic Black  Other Race | 276 (8.45%)  1199 (36.72%)  1220 (37.37%)  217 (6.65%) | 333 (9.89%)  1373 (40.77%)  825 (24.50%)  253 (7.51%) | 393 (11.66%)  1487 (44.12%)  518 (15.37%)  312 (9.26%) | 376 (11.16%)  1629 (48.34%)  332 (9.85%)  346 (10.27%) |  |
| HBA1C (%) | 5.37 ± 0.38 | 5.44 ± 0.37 | 5.51 ± 0.38 | 5.53 ± 0.38 | <0.001 |
| BMI (kg/m2) | 29.20 (26.80-33.30) | 29.67 (27.20-33.70) | 30.10 (27.40-34.00) | 30.85 (28.00-34.94) | <0.001 |
| FPG (mg/dl) | 90.07 ± 8.59 | 93.48 ± 8.92 | 95.82 ± 9.53 | 98.00 ± 10.21 | <0.001 |
| TC (mg/dl) | 179.00 ± 35.40 | 191.45 ± 37.63 | 200.44 ± 38.64 | 211.98 ± 41.33 | <0.001 |
| LDL (mg/dl) | 106.19 ± 30.14 | 117.94 ± 32.81 | 123.72 ± 34.39 | 125.78 ± 36.80 | <0.001 |
| HDL (mg/dl) | 59.84 ± 14.91 | 54.37 ± 14.11 | 50.62 ± 12.44 | 45.22 ± 11.85 | <0.001 |
| TG (mg/dl) | 61.00 (52.00-70.00) | 92.00 (84.00-101.00) | 127.0 (115.0-140.0) | 196.0 (169.0-235.0) | <0.001 |
| Stroke | 91 (2.79%) | 73 (2.17%) | 116 (3.44%) | 94 (2.79%) | 0.615 |
| SBP (mmHg) | 115.05 ± 21.47 | 118.83 ± 22.14 | 118.90 ± 23.40 | 119.00 ± 23.37 | <0.001 |
| DBP (mmHg) | 65.82 ± 13.90 | 67.62 ± 13.88 | 68.10 ± 14.89 | 68.60 ± 15.04 | <0.001 |
| Albumin(g/dl) | 4.14 ± 0.32 | 4.15 ± 0.34 | 4.15 ± 0.35 | 4.17 ± 0.38 | 0.005 |
| Creatinine(mg/dL) | 0.85 ± 0.34 | 0.87 ± 0.23 | 0.88 ± 0.37 | 0.90 ± 0.53 | 0.021 |
| Smoke | 1243 (38.07%) | 1426 (42.34%) | 1522 (45.16%) | 1654 (49.08%) | <0.001 |
| IFG | 363 (11.12%) | 666 (19.77%) | 955 (28.34%) | 1242 (36.85%) | <0.001 |
| CHD | 68 (2.08%) | 102 (3.03%) | 106 (3.15%) | 117 (3.47%) | 0.230 |
| HF | 49 (1.50%) | 61 (1.81%) | 78 (2.31%) | 103 (3.06%) | <0.001 |

Data are presented as number (%) or mean ± standard deviation (SD) or Median (Q1-Q3)

For continuous variables: P-value was by survey-weighted linear regression. For categorical variables: P-value was by survey-weighted Chi-square test.

TyG index, the triglyceride glucose index; BMI, body mass index; SBP, systolic blood pressure; DBP, diastolic blood pressure; LDL, low density lipoprotein; HDL, high density lipoprotein; TC, total cholesterol; TG, triglyceride; IFG, impaired fasting glucose; CHD, coronary heart disease; FPG, Fasting plasma glucose.
